# 2-[(5-Chloro­pyridin-2-yl­imino)­meth­yl]phenol

**DOI:** 10.1107/S2414314620000115

**Published:** 2020-01-31

**Authors:** Krishnasamy Mamallan, Sundaramoorthy Gomathi, Krishnan Soundararajan, Velusamy Sethuraman

**Affiliations:** a Department of Chemistry, Periyar Maniammai Institute of Science & Technology, Thanjavur 613 403, Tamil Nadu, India; University of Aberdeen, Scotland

**Keywords:** crystal structure, hydrogen bonds

## Abstract

In the title compound, C_12_H_9_ClN_2_O, the dihedral angle between the aromatic rings is 1.78 (4)° and an intra­molecular O—H⋯N hydrogen bond closes an *S*(6) ring. In the crystal, C—H⋯O and C—H⋯N hydrogen bonds connect the mol­ecules into [001] chains.

## Abstract 2

In the crystal, C—H⋯O and C—H⋯N hydrogen bonds connect the mol­ecules into [001] chains.

## Graphical abstract

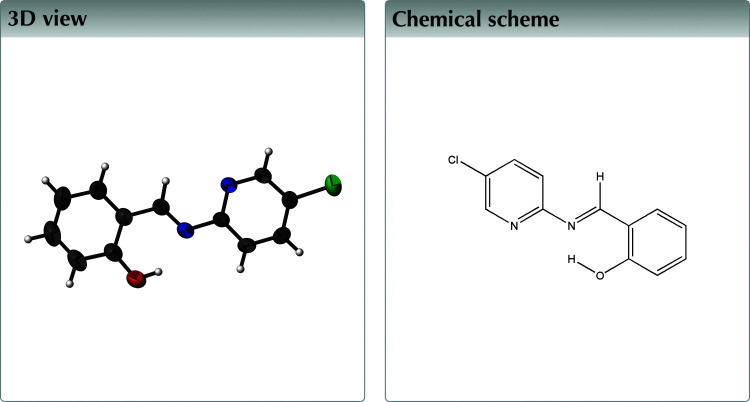

## Structure description

**Figure 1:**
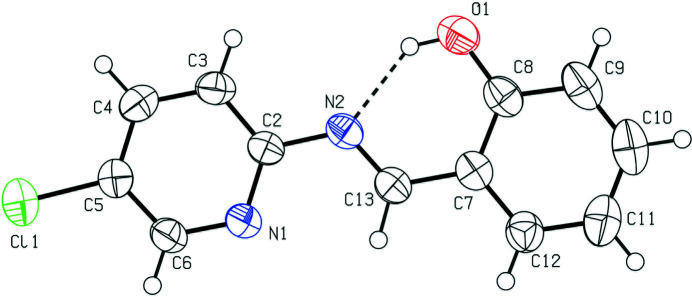
The mol­ecular structure of the title compound with displacement ellipsoids drawn at the 50% probability level. The O—H⋯N hydrogen bond is indicated by a dashed line.

**Figure 2:**
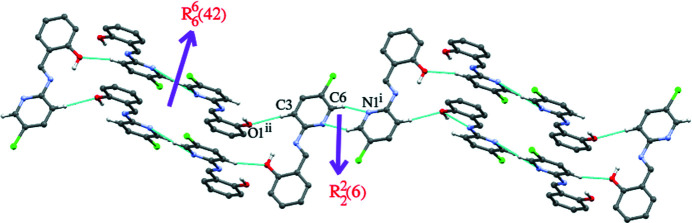
Supra­molecular strands formed by weak C—H⋯N and C—H⋯O hydrogen bonds. Symmetry codes: (i) 1 − *x*, 1 − *y*, −*z*; (ii) 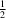 − *x*, − 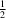 + *y*, 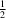 − *z*.

**Figure 3:**
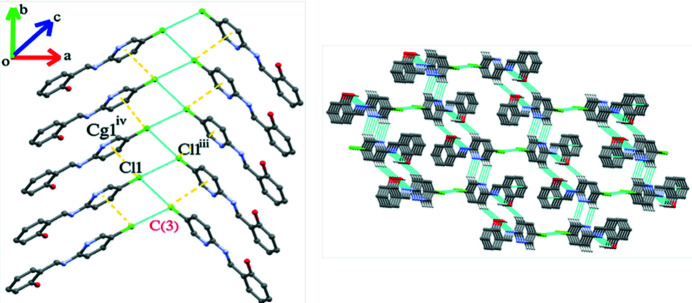
Left: Supra­molecular chain arising from Cl⋯Cl and C—Cl⋯π contacts. [Symmetry code: (iii) 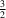 − *x*,- 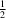 + *y*,1/2 − *z*; (iv) *x*,-1 + *y*, *z*]. Right: Honeycomb architecture formed by the weak non-covalent inter­actions.

The dihedral angle between the N1/C2–C6 pyridine ring and C7–C12 benzene ring is 1.78 (4)° and an intra­molecular O—H⋯N hydrogen bond closes an *S*(6) ring. The disposition of the aromatic rings is *trans* as indicated by the C2—N2—C13—C7 torsion angle of −179.7 (2)° (Fig. 1 ▸). In the crystal, centrosymmetric dimers linked by pairs of weak C6—H6⋯N1 hydrogen bonds (Table 1 ▸) generate 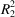 (6) loops. These dimers are linked by two pairs of C3—H3⋯O1 hydrogen bonds to form 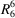 (42) loops (Fig. 2 ▸). These alternating loops lead to wave-like supra­molecular strands propagating along [001]. Inter­molecular Cl⋯Cl [3.476 (4)] and Cl⋯π [3.528 (4) Å] contacts slightly shorter than van der Waals separations are also observed (Fig. 3 ▸).

For the pharmaceutical behavior of Schiff bases, see: Mounika *et al.* (2010 ▸); Miri *et al.* (2013 ▸); Aboul-Fadl *et al.* (2003 ▸); Wei *et al.* (2006 ▸). For ring-opening reactions of pyrroles, see: Mannaert *et al.* (1997 ▸); . For halogen–halogen reactions, see: Pedireddi *et al.* (1994 ▸) and for halogen⋯π reactions, see: Rahman *et al.* (2003 ▸).

## Synthesis and crystallization

2-Amino-5-chloro­pyridine (1 mmol) and 2-hy­droxy benzaldehyde (1.2 mmol) were mixed in 20 ml of absolute ethanol with the addition of few drops of piperidine as catalyst. The mixture was refluxed for 5 h at 60–70°C. Colourless blocks of the title compound were obtained from the mother solution on cooling.

## Refinement

Crystal data, data collection and structure refinement details are summarized in Table 2 ▸.

## Supplementary Material

Crystal structure: contains datablock(s) global, I. DOI: 10.1107/S2414314620000115/hb4333sup1.cif

Structure factors: contains datablock(s) I. DOI: 10.1107/S2414314620000115/hb4333Isup2.hkl

Supporting information file. DOI: 10.1107/S2414314620000115/hb4333Isup3.cml

CCDC reference: 1975774

Additional supporting information:  crystallographic information; 3D view; checkCIF report

## Figures and Tables

**Table 1:** Hydrogen-bond geometry (Å, °)

*D*—H⋯*A*	*D*—H	H⋯*A*	*D*⋯*A*	*D*—H⋯*A*
O1—H8⋯N2	0.89 (5)	1.89 (5)	2.627 (4)	140 (4)
C3—H3⋯O1^i^	0.93	2.50	3.361 (5)	154
C6—H6⋯N1^ii^	0.93	2.59	3.365 (5)	141

Symmetry codes: (i) 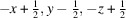 ; (ii) 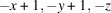 .

**Table 2:** Experimental details

Crystal data	
Chemical formula	C_12_H_9_ClN_2_O
*M*_r_	232.66
Crystal system, space group	Monoclinic, *P*2_1_/*n*
Temperature (K)	294
*a*, *b*, *c* (Å)	14.753 (12), 4.639 (3), 16.379 (16)
β (°)	105.35 (4)
*V* (Å^3^)	1081.0 (16)
*Z*	4
Radiation type	Mo *K*α
μ (mm^−1^)	0.33
Crystal size (mm)	0.14 × 0.12 × 0.09

Data collection	
Diffractometer	Bruker Kappa APEXII CCD
Absorption correction	Multi-scan (*SADABS*; Bruker, 2004 ▸)
*T*_min_, *T*_max_	0.955, 0.971
No. of measured, independent and observed [*I* > 2σ(*I*)] reflections	22920, 3033, 1878
*R*_int_	0.060
(sin θ/λ)_max_ (Å^−1^)	0.695

Refinement	
*R*[*F* ^2^ > 2σ(*F* ^2^)], *wR*(*F* ^2^), *S*	0.071, 0.219, 1.12
No. of reflections	3033
No. of parameters	154
H-atom treatment	H atoms treated by a mixture of independent and constrained refinement
Δρ_max_, Δρ_min_ (e Å^−3^)	0.36, −0.38

Computer programs: *APEX2* and *SAINT* (Bruker, 2004 ▸), *SHELXS97* and *SHELXL97* (Sheldrick, 2008 ▸), *Mercury* (Macrae *et al.*, 2008 ▸), *POV-RAY* (Cason, 2004 ▸), *PLATON* (Spek, 2020 ▸) and *publCIF* (Westrip, 2010 ▸).

## full crystallographic data

### Crystal data

**Table:**

C_12_H_9_ClN_2_O	*F*(000) = 480
*M**_r_* = 232.66	*D*_x_ = 1.430 Mg m^−^^3^
Monoclinic, *P*2_1_/*n*	Mo *K*α radiation, λ = 0.71076 Å
Hall symbol: -P 2yn	Cell parameters from 3033 reflections
*a* = 14.753 (12) Å	θ = 2.9–29.6°
*b* = 4.639 (3) Å	µ = 0.33 mm^−^^1^
*c* = 16.379 (16) Å	*T* = 294 K
β = 105.35 (4)°	Block, colourless
*V* = 1081.0 (16) Å^3^	0.14 × 0.12 × 0.09 mm
*Z* = 4	

### Data collection

**Table:**

Bruker Kappa APEXII CCD diffractometer	3033 independent reflections
Radiation source: fine-focus sealed tube	1878 reflections with *I* > 2σ(*I*)
Graphite monochromator	*R*_int_ = 0.060
ω and φ scan	θ_max_ = 29.6°, θ_min_ = 2.9°
Absorption correction: multi-scan (SADABS; Bruker, 2004)	*h* = −20→20
*T*_min_ = 0.955, *T*_max_ = 0.971	*k* = −6→6
22920 measured reflections	*l* = −22→22

### Refinement

**Table:**

Refinement on *F*^2^	Secondary atom site location: difference Fourier map
Least-squares matrix: full	Hydrogen site location: inferred from neighbouring sites
*R*[*F*^2^ > 2σ(*F*^2^)] = 0.071	H atoms treated by a mixture of independent and constrained refinement
*wR*(*F*^2^) = 0.219	*w* = 1/[σ^2^(*F*_o_^2^) + (0.0991*P*)^2^ + 0.6777*P*] where *P* = (*F*_o_^2^ + 2*F*_c_^2^)/3
*S* = 1.12	(Δ/σ)_max_ < 0.001
3033 reflections	Δρ_max_ = 0.36 e Å^−^^3^
154 parameters	Δρ_min_ = −0.38 e Å^−^^3^
0 restraints	Extinction correction: shelxl, FC^*^=KFC[1+0.001XFC^2^Λ^3^/SIN(2Θ)]^-1/4^
Primary atom site location: structure-invariant direct methods	Extinction coefficient: 0.096 (14)

### Special details

**Table:**

Geometry. Bond distances, angles etc. have been calculated using the rounded fractional coordinates. All su's are estimated from the variances of the (full) variance-covariance matrix. The cell esds are taken into account in the estimation of distances, angles and torsion angles
Refinement. Refinement on F^2^ for ALL reflections except those flagged by the user for potential systematic errors. Weighted R-factors wR and all goodnesses of fit S are based on F^2^, conventional R-factors R are based on F, with F set to zero for negative F^2^. The observed criterion of F^2^ > 2sigma(F^2^) is used only for calculating -R-factor-obs etc. and is not relevant to the choice of reflections for refinement. R-factors based on F^2^ are statistically about twice as large as those based on F, and R-factors based on ALL data will be even larger.

### Fractional atomic coordinates and isotropic or equivalent isotropic displacement parameters (Å^2^)

**Table:**

	*x*	*y*	*z*	*U*_iso_*/*U*_eq_	
Cl1	0.66137 (5)	0.10505 (17)	0.21103 (5)	0.0569 (3)	
O1	0.22420 (18)	1.2528 (6)	0.17721 (15)	0.0632 (9)	
N1	0.46749 (16)	0.6578 (5)	0.09207 (14)	0.0458 (7)	
N2	0.35223 (15)	0.9377 (5)	0.13429 (14)	0.0407 (7)	
C2	0.42598 (17)	0.7322 (5)	0.15259 (16)	0.0372 (8)	
C3	0.4511 (2)	0.6134 (6)	0.23285 (18)	0.0455 (9)	
C4	0.5240 (2)	0.4173 (6)	0.25253 (18)	0.0478 (9)	
C5	0.56824 (18)	0.3459 (6)	0.19071 (17)	0.0405 (8)	
C6	0.5382 (2)	0.4667 (7)	0.11164 (18)	0.0463 (9)	
C7	0.25370 (18)	1.2702 (6)	0.03971 (17)	0.0417 (8)	
C8	0.2054 (2)	1.3599 (6)	0.0985 (2)	0.0476 (9)	
C9	0.1342 (2)	1.5664 (7)	0.0744 (3)	0.0614 (13)	
C10	0.1122 (2)	1.6815 (8)	−0.0058 (3)	0.0657 (13)	
C11	0.1594 (2)	1.5959 (7)	−0.0644 (2)	0.0625 (11)	
C12	0.2300 (2)	1.3929 (6)	−0.04133 (19)	0.0505 (9)	
C13	0.32852 (19)	1.0574 (6)	0.06114 (18)	0.0413 (8)	
H3	0.41950	0.66460	0.27280	0.0550*	
H4	0.54300	0.33510	0.30610	0.0570*	
H6	0.56780	0.41440	0.07030	0.0560*	
H8	0.266 (3)	1.111 (10)	0.186 (3)	0.091 (15)*	
H9	0.10170	1.62620	0.11280	0.0740*	
H10	0.06480	1.81910	−0.02100	0.0790*	
H11	0.14380	1.67410	−0.11860	0.0750*	
H12	0.26230	1.33660	−0.08030	0.0610*	
H13	0.358 (2)	1.009 (7)	0.015 (2)	0.047 (8)*	

### Atomic displacement parameters (Å^2^)

**Table:**

	*U*^11^	*U*^22^	*U*^33^	*U*^12^	*U*^13^	*U*^23^
Cl1	0.0455 (5)	0.0518 (5)	0.0695 (6)	0.0066 (3)	0.0084 (3)	0.0004 (3)
O1	0.0711 (15)	0.0624 (15)	0.0660 (15)	0.0137 (12)	0.0356 (12)	0.0063 (12)
N1	0.0448 (12)	0.0539 (14)	0.0402 (12)	0.0083 (11)	0.0138 (10)	−0.0007 (10)
N2	0.0410 (12)	0.0376 (11)	0.0460 (13)	−0.0020 (9)	0.0158 (10)	−0.0057 (9)
C2	0.0376 (13)	0.0363 (13)	0.0387 (13)	−0.0046 (10)	0.0118 (10)	−0.0065 (10)
C3	0.0542 (16)	0.0452 (15)	0.0414 (14)	−0.0010 (12)	0.0202 (12)	−0.0029 (12)
C4	0.0560 (16)	0.0466 (15)	0.0396 (14)	−0.0020 (13)	0.0107 (12)	0.0037 (12)
C5	0.0360 (12)	0.0377 (13)	0.0451 (15)	−0.0032 (10)	0.0060 (11)	−0.0027 (11)
C6	0.0453 (15)	0.0528 (16)	0.0426 (15)	0.0070 (12)	0.0147 (12)	−0.0052 (12)
C7	0.0391 (13)	0.0357 (13)	0.0499 (15)	−0.0057 (11)	0.0110 (11)	−0.0058 (11)
C8	0.0404 (14)	0.0424 (15)	0.0636 (18)	−0.0047 (11)	0.0200 (13)	−0.0048 (13)
C9	0.0480 (16)	0.0505 (17)	0.092 (3)	0.0043 (14)	0.0296 (16)	−0.0033 (17)
C10	0.0445 (16)	0.0496 (17)	0.097 (3)	0.0057 (14)	0.0080 (17)	0.0017 (18)
C11	0.0592 (19)	0.0510 (17)	0.066 (2)	0.0043 (15)	−0.0034 (16)	0.0012 (15)
C12	0.0520 (16)	0.0459 (15)	0.0513 (16)	0.0032 (13)	0.0095 (13)	−0.0043 (13)
C13	0.0405 (13)	0.0388 (13)	0.0453 (15)	−0.0025 (11)	0.0125 (11)	−0.0055 (11)

### Geometric parameters (Å, º)

**Table:**

Cl1—C5	1.733 (3)	C7—C8	1.404 (4)
O1—C8	1.340 (4)	C8—C9	1.399 (5)
O1—H8	0.89 (5)	C9—C10	1.375 (7)
N1—C6	1.342 (4)	C10—C11	1.385 (5)
N1—C2	1.341 (4)	C11—C12	1.381 (5)
N2—C13	1.282 (4)	C3—H3	0.9300
N2—C2	1.418 (4)	C4—H4	0.9300
C2—C3	1.383 (4)	C6—H6	0.9300
C3—C4	1.380 (4)	C9—H9	0.9300
C4—C5	1.382 (4)	C10—H10	0.9300
C5—C6	1.373 (4)	C11—H11	0.9300
C7—C12	1.401 (4)	C12—H12	0.9300
C7—C13	1.453 (4)	C13—H13	0.99 (3)
			
Cl1···C6^i^	3.627 (5)	C8···N2^vi^	3.398 (5)
Cl1···Cl1^ii^	3.476 (4)	C8···C2^vi^	3.582 (5)
Cl1···H11^iii^	3.1500	C10···C13^vi^	3.546 (6)
O1···N2	2.627 (4)	C10···C7^vi^	3.398 (6)
O1···C3^iv^	3.361 (5)	C11···C13^vi^	3.510 (5)
O1···H3^iv^	2.5000	C13···N1^vi^	3.416 (5)
O1···H8^iv^	2.76 (5)	C13···C6^vi^	3.536 (5)
N1···C13^i^	3.416 (5)	C13···C10^i^	3.546 (6)
N1···C6^v^	3.365 (5)	C13···C11^i^	3.510 (5)
N2···O1	2.627 (4)	C4···H11^viii^	3.0300
N2···C8^i^	3.398 (5)	C6···H6^v^	3.0300
N1···H6^v^	2.5900	C11···H4^ix^	3.0800
N1···H13	2.40 (3)	C13···H8	2.47 (5)
N2···H8	1.89 (5)	H3···O1^vii^	2.5000
C2···C5^vi^	3.494 (5)	H4···C11^iii^	3.0800
C2···C7^i^	3.462 (5)	H6···N1^v^	2.5900
C2···C8^i^	3.582 (5)	H6···C6^v^	3.0300
C3···O1^vii^	3.361 (5)	H8···N2	1.89 (5)
C5···C2^i^	3.494 (5)	H8···C13	2.47 (5)
C6···C13^i^	3.536 (5)	H8···O1^vii^	2.76 (5)
C6···Cl1^vi^	3.627 (5)	H11···Cl1^ix^	3.1500
C6···N1^v^	3.365 (5)	H11···C4^x^	3.0300
C6···C6^v^	3.544 (5)	H12···H13	2.3600
C7···C2^vi^	3.462 (5)	H13···N1	2.40 (3)
C7···C10^i^	3.398 (6)	H13···H12	2.3600
			
C8—O1—H8	113 (3)	C10—C11—C12	119.2 (3)
C2—N1—C6	118.2 (2)	C7—C12—C11	121.2 (3)
C2—N2—C13	119.5 (2)	N2—C13—C7	121.5 (3)
N1—C2—N2	119.5 (2)	C2—C3—H3	121.00
N1—C2—C3	122.7 (2)	C4—C3—H3	121.00
N2—C2—C3	117.8 (2)	C3—C4—H4	121.00
C2—C3—C4	118.8 (3)	C5—C4—H4	121.00
C3—C4—C5	118.5 (3)	N1—C6—H6	119.00
Cl1—C5—C6	119.1 (2)	C5—C6—H6	119.00
C4—C5—C6	119.8 (3)	C8—C9—H9	120.00
Cl1—C5—C4	121.2 (2)	C10—C9—H9	120.00
N1—C6—C5	122.1 (3)	C9—C10—H10	120.00
C8—C7—C12	119.0 (3)	C11—C10—H10	119.00
C12—C7—C13	119.2 (3)	C10—C11—H11	120.00
C8—C7—C13	121.8 (3)	C12—C11—H11	120.00
O1—C8—C7	122.4 (3)	C7—C12—H12	119.00
C7—C8—C9	119.3 (3)	C11—C12—H12	119.00
O1—C8—C9	118.3 (3)	N2—C13—H13	123.2 (19)
C8—C9—C10	120.4 (3)	C7—C13—H13	115.3 (19)
C9—C10—C11	121.1 (3)		
			
C6—N1—C2—N2	178.8 (2)	C12—C7—C8—O1	−179.9 (3)
C6—N1—C2—C3	−2.1 (4)	C12—C7—C8—C9	0.8 (4)
C2—N1—C6—C5	0.4 (4)	C13—C7—C8—O1	−0.9 (4)
C13—N2—C2—N1	−2.5 (4)	C13—C7—C8—C9	179.8 (3)
C13—N2—C2—C3	178.4 (3)	C8—C7—C12—C11	−1.0 (4)
C2—N2—C13—C7	−179.7 (2)	C13—C7—C12—C11	−180.0 (3)
N1—C2—C3—C4	2.2 (4)	C8—C7—C13—N2	1.5 (4)
N2—C2—C3—C4	−178.7 (3)	C12—C7—C13—N2	−179.5 (3)
C2—C3—C4—C5	−0.6 (4)	O1—C8—C9—C10	−179.7 (3)
C3—C4—C5—Cl1	179.1 (2)	C7—C8—C9—C10	−0.4 (5)
C3—C4—C5—C6	−1.0 (4)	C8—C9—C10—C11	0.2 (5)
Cl1—C5—C6—N1	−179.0 (2)	C9—C10—C11—C12	−0.4 (5)
C4—C5—C6—N1	1.1 (5)	C10—C11—C12—C7	0.8 (5)

Symmetry codes: (i) *x*, *y*−1, *z*; (ii) −*x*+3/2, *y*+1/2, −*z*+1/2; (iii) *x*+1/2, −*y*+3/2, *z*+1/2; (iv) −*x*+1/2, *y*+1/2, −*z*+1/2; (v) −*x*+1, −*y*+1, −*z*; (vi) *x*, *y*+1, *z*; (vii) −*x*+1/2, *y*−1/2, −*z*+1/2; (viii) *x*+1/2, −*y*+5/2, *z*+1/2; (ix) *x*−1/2, −*y*+3/2, *z*−1/2; (x) *x*−1/2, −*y*+5/2, *z*−1/2.

### Hydrogen-bond geometry (Å, º)

**Table:**

*D*—H···*A*	*D*—H	H···*A*	*D*···*A*	*D*—H···*A*
O1—H8···N2	0.89 (5)	1.89 (5)	2.627 (4)	140 (4)
C3—H3···O1^vii^	0.93	2.50	3.361 (5)	154
C6—H6···N1^v^	0.93	2.59	3.365 (5)	141

Symmetry codes: (v) −*x*+1, −*y*+1, −*z*; (vii) −*x*+1/2, *y*−1/2, −*z*+1/2.
